# The analysis of relapse-free survival curves: implications for evaluating intensive systemic adjuvant treatment regimens for breast cancer

**DOI:** 10.1038/sj.bjc.6602267

**Published:** 2005-01-14

**Authors:** R S Day, S E Shackney, W P Peters

**Affiliations:** 1 Department of Biostatistics, University of Pittsburgh, Pittsburgh, PA, USA; 2 Laboratory of Cancer Cell Biology and Genetics, Department of Human Oncology, Allegheny Singer Research Institute, Allegheny General Hospital, Pittsburgh, 320 East North Avenue, PA 15212, USA; 3 Adherex Technologies, Durham, NC, USA; 4 Department of Human Oncology, Allegheny General Hospital, and Laboratory of Cancer Cell Biology and Genetics, Allegheny-Singer Research Institute

**Keywords:** breast cancer, high-dose therapy, mathematical model

## Abstract

Results of adjuvant dose intensification studies in patients with localised breast cancer have raised questions regarding the clinical usefulness of this treatment strategy. Here, we develop and fit a natural history model for the time to clinical tumour recurrence as a function of the number of involved lymph nodes, and derive plausible predictions of the effects of dose intensification under various conditions. The time to tumour recurrence is assumed to depend on the residual postoperative micrometastatic burden of tumour, the fractional reduction of residual tumour burden (RTB) by treatment, and the rate of regrowth of the RTB to a clinically detectable size. It is assumed that a proportion of micrometastatic tumours are unresponsive to adjuvant chemotherapy even at maximal dose intensity. Data fitted included the San Antonio Cancer Institute (SACI) database of untreated patients, and CALGB #9082, a study comparing a highly intensive and moderately intensity adjuvant regimen in patients with 10+ positive axillary nodes. The proportion of tumours unresponsive to maximally intensive adjuvant treatment is estimated to be 48% (29–67%). The estimated log kill for intermediate-dose therapy from CALGB #9082 was 6.5 logs, compared with 9 logs or greater for high-dose therapy. The model is consistent with a modest but nonnegligible advantage of dose intensification compared with standard therapies in patients with sensitive tumours who have 10+ positive axillary nodes, and suggests that much of this clinical benefit could be achieved using intermediate levels of treatment intensification. The model further suggests that, in patients with fewer than 10 involved axillary nodes, any advantage of treatment intensification over standard therapy would be much reduced, because in patients with smaller tumour burdens of sensitive tumour, a larger proportion of cures achievable with intensified therapy could be achieved as well with standard therapy.

Several large prospective randomised clinical trials have suggested a modest increase in relapse-free survival (RFS) with chemotherapeutic treatment intensification in patients with 10 or more positive axillary nodes, while others have not (Roche *et al*, 2003; Rodenhuis *et al*, 2003; Tallman *et al*, 2003; Zander *et al*, 2004). Thus, the clinical circumstances under which patients might benefit from this effect remain to be identified. To guide design choices for future clinical trials of dose intensification in the adjuvant therapy of human breast cancer, we have developed a mathematical model to extract information regarding the natural history of breast cancers and their sensitivity to chemotherapy from clinical RFS curves.

The time to postoperative recurrence of a tumour in an untreated patient is determined by: (1) the size of the subclinical residual tumour burden (RTB) following surgery and (2) the growth curve trajectory for the residual tumour in that patient (see Figure 1). Therefore, from the RFS curve for a cohort of untreated patients, it might be possible to estimate the distribution of RTB and the distribution of growth trajectories. In patients who receive systemic adjuvant treatment, the RTB after treatment depends, at minimum, on three things: the RTB after surgery but before treatment, the dose and schedule of the treatment, and the sensitivity of the cancer cells to the treatment. Therefore, from the RFS curve for treated patients, it might be possible to estimate the response of tumour to adjuvant therapy. (Here the RFS estimate treats deaths without prior recurrence as censored, because the model is intended to infer the relationship between underlying tumour biology and clinical tumour behaviour.)

Many elements of earlier models have been incorporated into the model proposed in this paper. The model assumes Gompertzian growth (Laird, 1969; Norton, 1988), and simultaneously fits the parameters of the RTB distribution and the micrometastatic tumour growth rate distribution to the RFS curve (Gregory *et al*, 1991). To model the effects of systemic therapy, we have adopted the log-kill hypothesis (Skipper *et al*, 1964, 1965), with the additional assumption that there may be a distinct subset of patients with tumours that are absolutely resistant to even the most intensive regimens that can be administered clinically. This assumption provides the simplest model allowing for both highly responsive and highly unresponsive patients.

The analysis leads to quantitative estimates of the RTB distribution, the subclinical tumour growth rate distribution, the proportion of patients with resistant tumours, and the log kill as a function of treatment intensity. The impact of simplifying assumptions and other potential sources of error on the estimates is substantial (see Appendix A1), but the broad insights are reasonably robust. Therefore, the goal of this investigation is limited to providing plausible *qualitative* explanations for the clinical trial results that can stimulate hypotheses to guide the performance of overviews and the design of future clinical trials.

## MATERIALS AND METHODS

### Model

**Figure 1:**
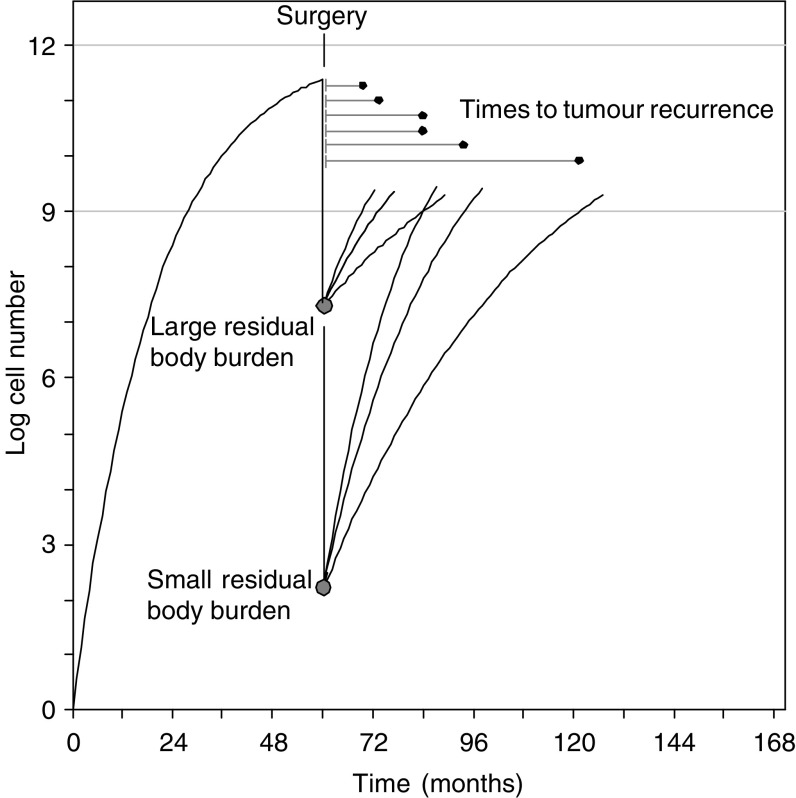
Schematic representation of the factors that affect the postoperative time delay to clinical detection of micrometastatic disease, and their relation to RFS curves in patients with stage I–III breast cancer. The log of tumour cell number is plotted as a function of time. Tumours are assumed to undergo growth retardation as they enlarge. The threshold for clinical detection of disease is assumed to be ∼1 × 10^9^ cells (approximately 1 g of tumour, which may occupy 0.5–10 cm^3^, depending on stromal components and oedema). Large postoperative burdens of residual micrometastatic disease (say >10^7^ cells) are likely to recur early (say, within 1–2 years). Small micrometastatic tumour burdens (<1 × 10^3^ cells) are likely to cross the threshold of clinical detection later than large micrometastatic tumour burdens. Since the growth rate characteristics of micrometastatic tumours of comparable size may vary from patient to patient, it is to be expected that there will be overlap in times to recurrence among patients with small and large micrometastatic tumour burdens, particularly among patients with intermediate micrometastatic tumour burdens and intermediate subclinical growth rates. If micrometastases can lie dormant for long periods and then commence to grow rapidly, their behaviour would be indistinguishable in the present model from continuously slowly growing micrometastases that recur clinically at comparable times.

The time to tumour recurrence is treated as a deterministic function of the residual postoperative micrometastatic tumour burden and the growth path of micrometastatic disease, each of which are random and independent (see Figure 1). The convolution of these two distributions generates RFS curves that are fitted to clinical data. Mathematical details and rationales for key modelling decisions are provided briefly in Appendix A1, and extensively in a technical supplement available at the website www.oncotcap.pitt.edu/docs/rec urrence-overview.

The fitting of RFS curves, even in the absence of systemic treatment and even with large sample sizes, poses serious identifiability problems; that is, even a relatively simple model requires more parameters than can be simultaneously and accurately estimated. To reduce the number of parameters and obtain reasonably stable estimates, strong assumptions have been made. The two random quantities, the RTB and the growth rate, are assumed independent of each other. The growth of micrometastases is assumed to follow the Gompertz growth model. Certain parameters are assigned fixed values, including the Gompertz plateau (10^13^ cells), the deceleration rate (1), and a minimum value for the time from one cell to recurrence (6 months). The two parameters governing the RTB are assumed to change linearly with increasing numbers of involved axillary nodes (as ordered categories).

The cytoreductive effects of a chemotherapeutic drug regimen are modelled by shifting the tumour burden distribution downward by a fixed proportion, in accordance with the log-kill hypothesis. Intratumour heterogeneity is not modelled. Instead, the ‘log kill’ represents a net fractional reduction in tumour cell tumour burden across tumour cell subpopulations and over the entire course of adjuvant treatment. Effects of systemic therapy on growth rates of tumour cells are assumed to be transient (lasting days to weeks) and are not taken into account. The magnitude of the log-kill shift is assumed to vary with treatment intensity, but not necessarily in proportion to dose. Intertumour heterogeneity in responsiveness to chemotherapy is represented as a two-point distribution, with one point anchored at log kill=zero. Thus, it is assumed that a proportion of tumours is unresponsive even to maximally intensive therapy. This proportion of absolutely resistant tumours is treated as another model parameter to be fitted. The apparent conflict between this assumption and observations of partial or complete responses is discussed in Appendix A1.

### Data sets

Data from the San Antonio Cancer Institute (SACI) on 3217 postoperative breast cancer patients with stage I–III disease who received no systemic chemotherapy or hormonal therapy postoperatively were kindly provided by Dr Gary Clark. These data were used to estimate the distribution of RTBs of tumour and the distribution of growth rate parameters in untreated patients. Deaths prior to recurrence were treated as censored. To reduce computational burden from numerical integration, the fitting was applied to a subsample consisting of 15.4% (400/2595) randomly sampled from the 81% who were node-negative, together with all (622) node-positive patients. The results were compared to the omitted patients and to a similar data set, NSABP B04, obtained through the generosity of Dr John Bryant.

In 1999, CALGB #9082, an intergroup randomised study of patients with 10 or more positive nodes treated with either a highly dose-intensive or intermediately intensive chemotherapeutic regimen was presented and published in abstract form (Peters *et al*, 1999). At that time, the median time on study was roughly 3 years. The findings after 5 years of follow-up were updated at a National of Institutes of Health-sponsored consensus conference in November of 2000, including ‘RFS’ data in which deaths without recurrence were treated as censored (Peters *et al*, 2000). Both the high- and intermediate–dose (ID) RFS curves exhibited reverse sigmoid shapes, decreasing slowly during the first 6–9 months, more rapidly over the next 4–5 years, and more slowly again thereafter. The high- and ID RFS curves were nearly superimposable during the first 6 months of follow-up, and then diverged. By the second year of follow-up, the difference between the two curves appeared to stabilise, and was maintained between 7 and 12% thereafter. This is discussed further below.

After 5 years of follow–up, the fraction of high-dose patients free of recurrence was approximately 0.65, and that of ID patients was approximately 0.57. Owing to 7% of the early treatment-related early deaths on the high-dose arm, comparability of the two RFS curves is of course not assured. In principle, it is possible that in the absence of fatal complications, these patients would have all relapsed in the first 5 years. This would yield a high-dose arm 5-year RFS rate of 60% instead of 65%, eliminating most of the observed difference in the arms. If instead, those susceptible to fatalities would have been at higher risk for early recurrence with an odds ratio of 2, then the combined high-dose RFS rate at 5 years would be 63.5%, little different from the observed 65%. Therefore, we are fairly comfortable in tentatively neglecting this issue.

These data points from the published abstract were used to estimate the resistant fraction, as detailed below. Recently published high-dose RFS curves are similar to those of CALGB #9082. The fractions of high-dose patients free of recurrence at 5 years are in the range of 0.58–0.62 (Roche *et al*, 2003; Rodenhuis *et al*, 2003; Tallman *et al*, 2003).

### Strategy for model fitting

The initial step was to jointly estimate the shared growth distribution and the individual residual tumour distributions for four axillary nodal status groups, defined, respectively, as patients with zero, 1–3, 4–9, or 10+ positive axillary nodes, using the SACI database. The per cent of patients with absolutely resistant disease was estimated by fitting the 5-year RFS rate from the high-dose arm of the CALGB #9082, assuming a log kill of 9 for the sensitive patients to represent the maximal log kill. The log kill for the ID arm was then estimated assuming the same fixed per cent of absolutely resistant tumours. A representative log kill for standard dose was taken to be roughly the median of fitted values from a set of 5-year RFS rates from published studies. The identity of constituent chemotherapy agents was ignored for this purpose. Finally, implications were derived assuming that the estimated proportion of patients with resistant tumours is independent of the degree of lymph node involvement. Where possible, sensitivity analysis relative to the assumptions was performed.

### Parameter estimation

Estimation was based on the method of maximum likelihood. Likelihoods were computed by numerical integration, specifically the adaptive 15-point Gauss–Kronrod quadrature as implemented in Splus®. Maxima were calculated using the Newton–Gauss–Seidel method. No evidence of multimodality was found. Confidence intervals for parameters were based on the Hessian matrices, and the delta method was used to obtain variance estimates for the plateau as a function of the parameters.

## RESULTS

### RTB in relation to the number of involved axillary nodes

**Figure 2:**
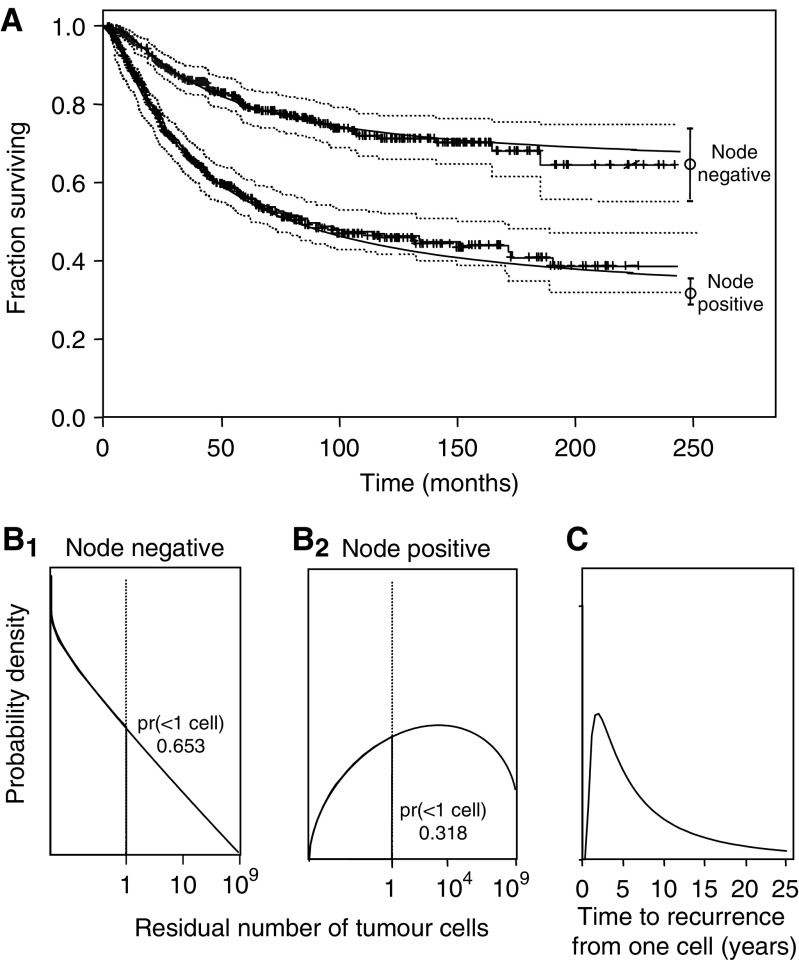
The model was applied to RFS curves from the San Antonio Database for node-negative and node-positive breast cancer patients. (**A**) Actual node-negative and node-positive RFS curves (upper and lower thin-lined solid curves, respectively) are shown with censored patients marked by crosses, and are bracketed by dotted curves representing 95% confidence limits. Corresponding modelled RFS curves are also shown (thick-lined solid curves), with means at infinite follow-up and 95% confidence limits for the fitted curves shown to the right of each curve. (**B_1_**) Fitted distribution of residual micrometastatic cell burden for node-negative patients. Grey zone indicates the portion of the distribution that represents the proportion of patients with <1 micrometastatic cell (pr), which was 0.65. (**B_2_**) Fitted distribution of residual micrometastatic cell burden for node-positive patients. The grey zone indicates the portion (32%) of the distribution corresponding to patients with <1 micrometastatic cell (pr). (**C**) Fitted distribution of times to recurrence from a single cell. It is assumed to be the same for all axillary nodal groups.

**Figure 3:**
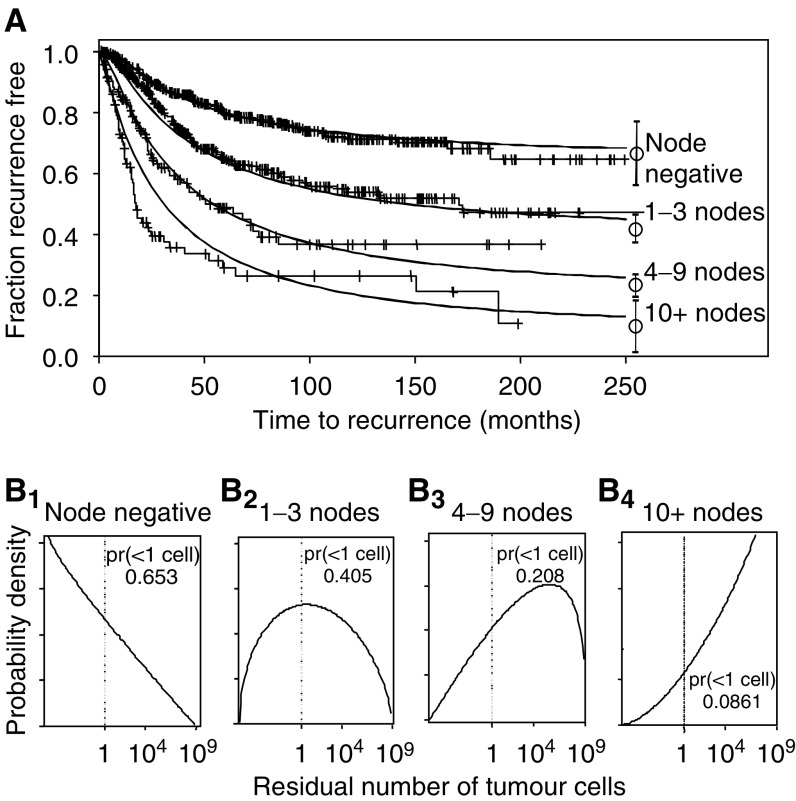
The model was applied to RFS curves from the San Antonio Database for four different subsets of breast cancer by axillary nodal status: node negative, 1–3 positive nodes, 4–9 positive nodes, and 10+ positive nodes. (**A**) Actual RFS curves (thin-lined solid curves) are shown with censored patients marked by crosses. Corresponding modelled RFS curves are also shown (thick-lined solid curves), with means at infinite follow-up and 95% confidence limits for the fitted curves shown to the right of each curve. (**B_1_–B_4_**) Fitted distributions of residual micrometastatic cell burden for subsets of patients modelled in (**A**). The grey zones indicate the portions of the distribution that represent patients with <1 micrometastatic cell (pr). The fitted distribution of times to recurrence from a single cell is identical to Figure 2C.

Model-predicted RFS distributions for node-negative and node-positive patients are compared to the estimated SACI RFS distributions (Figure 2). The node-positive model-based curve is a mixture of curves for the three node-positive subgroups, weighted by the SACI node distribution. The estimated proportion of patients rendered relapse free by surgery (RFS curve at *T*=∞) is 65% (56–75%) for node-negative and 32% (29–35%) for node-positive patients (95% confidence intervals within parentheses). The RFS estimates for each of the four node groups are shown in Figure 3. The family of fitted curves is consistent with a direct relationship between RTB and the number of involved axillary nodes. The estimated proportions of patients rendered relapse free by surgery alone were 65, 41, 21, and 9% for patients with no axillary node involvement, 1–3 positive nodes, 4–9 positive nodes, and 10 or more positive nodes, respectively. The goodness of fit for 10+ positive nodes is adequate for the purpose (*P*=0.17 using log-rank test *vs* simulated data with *N*=10 000).

### Responsiveness and resistance to chemotherapy in breast cancer patients with 10 or more positive axillary nodes

**Figure 4:**
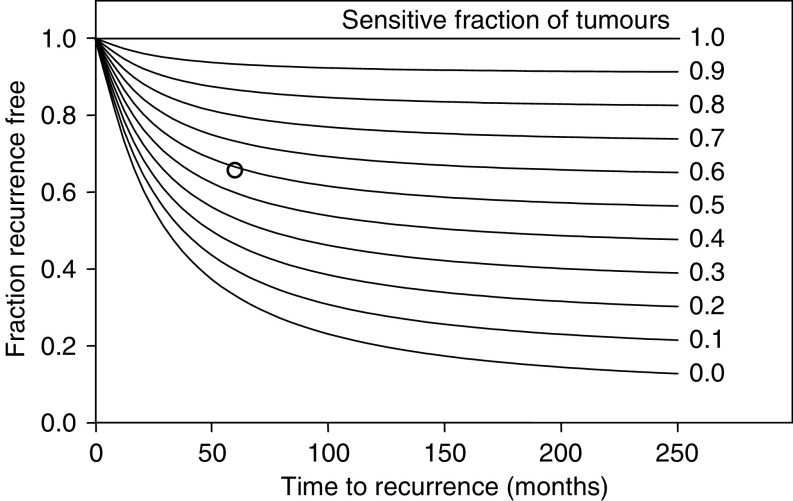
Simulation of RFS in patients with 10 or more positive axillary nodes following maximally intensive therapy, assuming different proportions of patients with sensitive tumours ranging from 0 to 1.0, in 0.1 increments. Treatment of maximally tolerated intensity is assumed to produce a 9 log cell kill in sensitive tumours, and is assumed to produce no effect on resistant tumours. For reference, the value for RFS at 5 years following treatment of maximal intensity using the Peters regimen (Peters *et al*, 2000) is shown (open circle), corresponding to an estimated probability of a chemosensitive tumour equal to 0.48.

The distributions of RTB and growth rate previously estimated for untreated patients with 10 or more positive axillary nodes (Figure 3) were combined with postulated values for the proportion of patients with absolutely resistant tumours (Figure 4). From CALGB #9082 (Peters *et al*, 2000), the RFS proportion after 5 years was estimated to be 0.65. RFS curves were calculated as a function of the fraction of absolutely resistant tumours applied to CALGB #9082. With the assumption that the log kill in sensitive tumours is maximal (9 or greater), the maximum-likelihood estimate for the proportion of sensitive tumours is 48% (95% confidence interval=29–67% based on sampling uncertainty in the 5-year point estimate from CALGB 9082).

**Figure 5:**
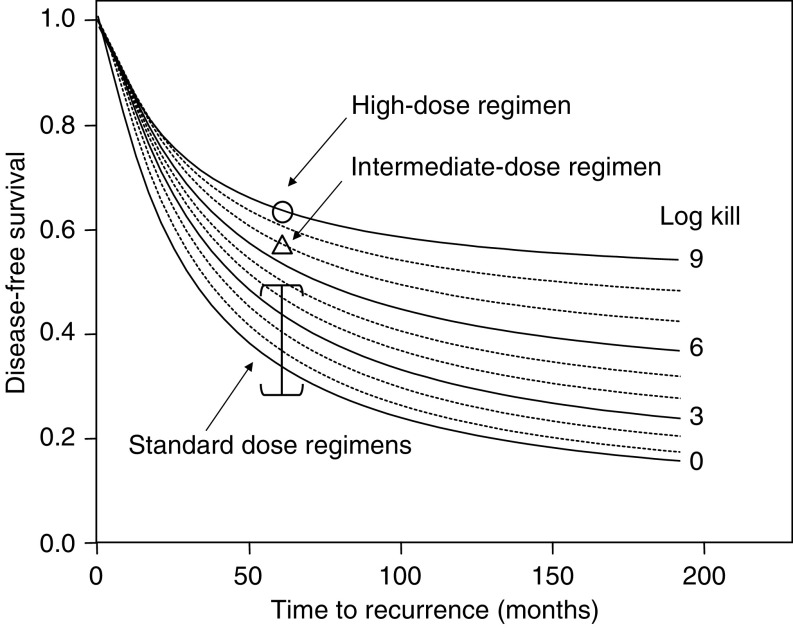
Simulation of RFS in patients with 10 or more positive axillary nodes assuming different overall log kills per treatment regimen ranging from 0 to 1.0, in 0.1 increments. The proportion of patients with sensitive tumours is assumed to be 0.48 Treatment of maximally tolerated intensity is assumed to produce a 9 log cell kill in sensitive tumours, and is assumed to produce no effect on resistant tumours. For reference, the value for RFS at 5 years following treatment of maximal intensity using the Peters regimen (*5*) is shown (open circle). The value for RFS at 5 years for the Peters ID regimen (*5*) is shown as an open triangle. A range of estimated values of RFS at 5 years taken from published studies (see Table 1) is shown as an bounded vertical line.

To estimate the log kill of therapy of ID intensity on sensitive tumours in the Peters study, this estimate of 48% was used to derive a family of RFS curves as a function of the log kill (Figure 5). As the log kill increases, the proportion of patients who remain relapse free over the long-term increases, but approaches a limit imposed by the fraction of absolutely resistant tumours. These curves, particularly those ranging from 3 to 9 logs of cell kill, exhibit minimal differences during the first year of follow-up, with larger differences becoming more apparent at later times, in keeping with clinical findings (Peters *et al*, 2000; Roche *et al*, 2003; Rodenhuis *et al*, 2003; Tallman *et al*, 2003). The 5-year RFS estimate for the Peters ID regimen (Peters *et al*, 2000) is shown as a triangle. The maximum-likelihood estimate of the log kill for the ID regimen is 6.9 logs.

The 5-year RFS points for high-dose regimens from more recently published studies (Roche *et al*, 2003; Rodenhuis *et al*, 2003; Tallman *et al*, 2003) are and 55%. The log kills for other high-dose regimens (Roche *et al*, 2003; Rodenhuis *et al*, 2003; Tallman *et al*, 2003) on sensitive tumours are estimated to range between 7 and 9 logs, assuming that the sensitive tumour fraction is again 48%.

Historical and recently published data on the fractions of patients with 10 or more positive axillary nodes, who survive relapse free 5 years after *standard-dose therapy* are summarised in Table 1. In most studies, RFS at 5 years was in the range of 20–40% (Rodenhuis *et al*, 2003; Tallman *et al*, 2003). Figure 5 suggests that the log kill for standard-dose therapy in sensitive patients with 10 or more positive nodes is in the range of 2–4 logs, with an upper limit of about 5 logs.

### Treatment intensification in patients with fewer than 10 positive axillary nodes

**Figure 6:**
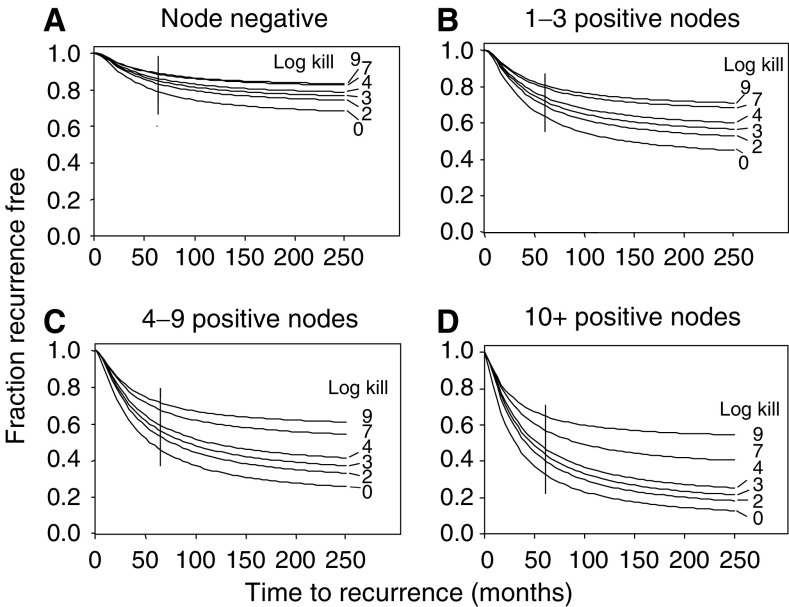
Modelled effects of standard therapy (assumed to produce –4 logs of cell kill, shown as shaded region), therapy of intermediate intensity (producing a 7 log cell kill), and therapy of maximal intensity (producing a 9 log cell kill) in different subsets of patients by nodal status. (**A**) Node-negative patients. (**B**) Patients with 1–3 positive nodes. (**C**) Patients with 4–9 positive nodes. (**D**) Patients with 10+ positive nodes.

To make predictions for treatment of patients with fewer than 10 positive axillary nodes, we now add the assumption that the fraction of sensitive micrometastatic tumours changes negligibly with the degree of nodal involvement. Figure 6 compares model predictions of the effects of varying the degree of the log kill on RFS curves, for patient groups defined by the number of positive axillary nodes. In node-negative patients, the predicted benefit of 2 to 4 logs of cell kill (standard therapy) is modest but real. This accords with the findings of the 2002 overview update (Early Breast Cancer Trialists' Collaborative Group, 2004). The 10-year survival estimate in node-negative patients was increased by adjuvant chemotherapy from 71 to 78%. The predicted additional increase in RFS from increasing the log kill, even to a very high level, is small. Moving to the other panels, we see that the differences in RFS between intensive and standard-dose therapy decrease dramatically as the number of positive nodes decreases.

**Figure 7:**
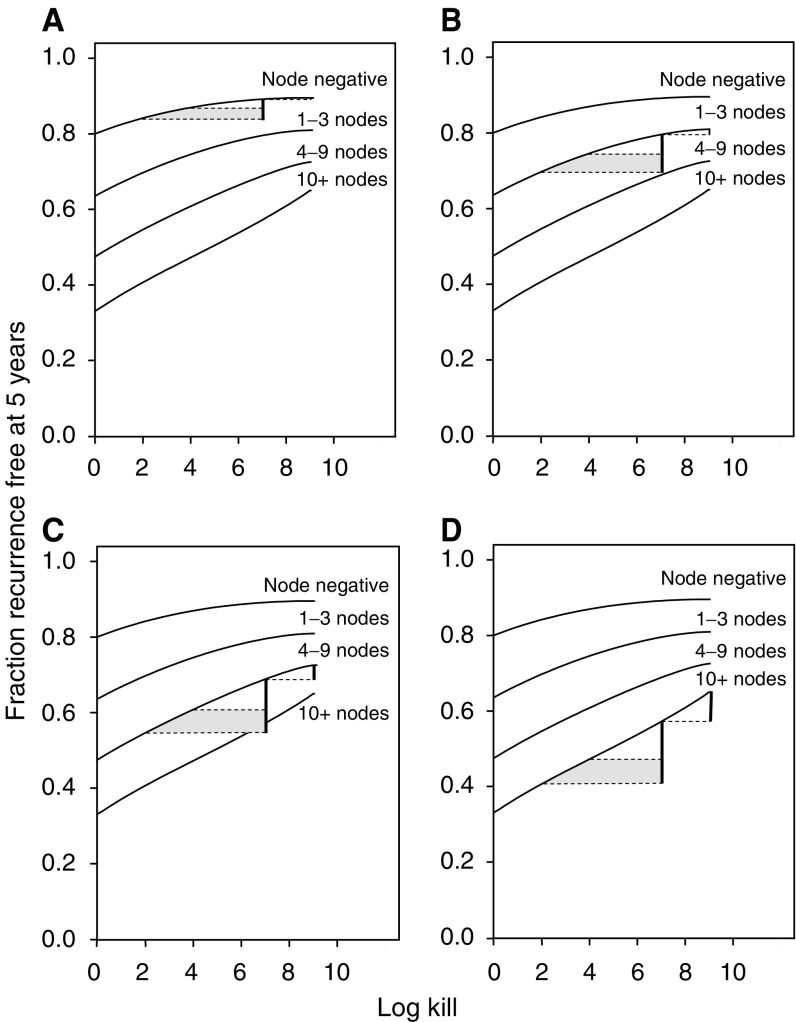
Incremental RFS benefit for adjuvant therapy of intermediate intensity and maximal intensity (heavy vertical line) in subsets of patients by nodal status. A 7 log kill is assumed for therapy of intermediate intensity, and therapy of maximal intensity is assigned a 9 log kill. A sensitive fraction of tumours of 0.48 is assumed. (**A**) Node-negative patients. (**B**) Patients with 1–3 positive nodes. (**C**) Patients with 4–9 positive nodes. (**D**) Patients with 10+ positive nodes.

**Figure 8:**
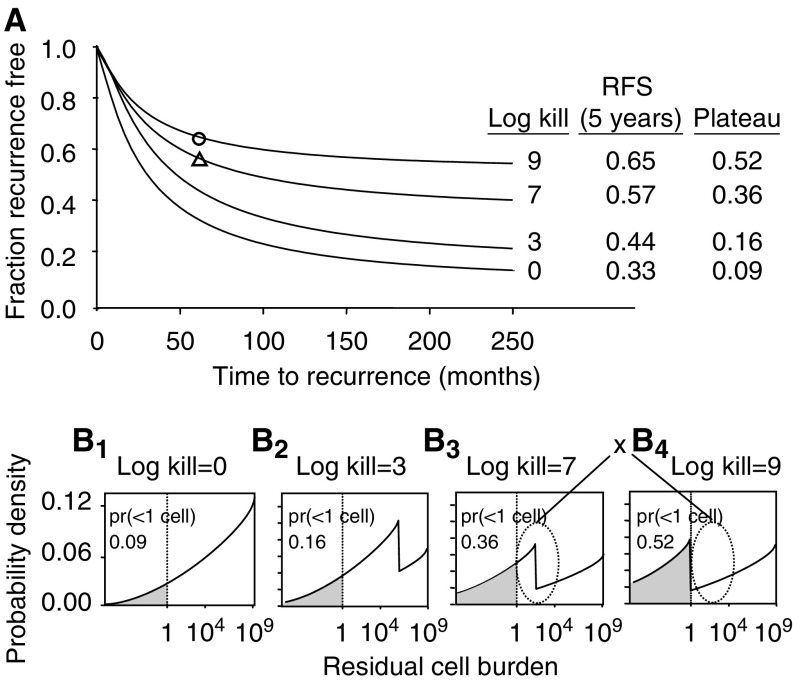
(**A**) A comparison of modelled RFS curves in patients with 10+ positive axillary nodes, who were untreated or were given treatments that produced 3, 7, or 9 logs of cell kill. For reference, actual 5-year RFSs are shown after treatment with the Peters maximally tolerated regimen (open circle) and the intermediate regimen (open triangle). (**B_1_–B_4_**) Modelled distributions of micrometastatic residual cell numbers after surgery and either no systemic treatment (**B_1_**) or systemic therapies that produce 3 logs (**B_2_**), 7 logs (**B_3_**), or 9 logs (**B_4_**) of cell kill. Shaded areas in each distribution reflect the probabilities that less than one viable tumour cell survived after treatment. Oval regions marked by ‘x’ highlight the differences in the residual body burden distribution as a consequence of a 7 log kill *vs* a 9 log kill. For discussion, see text.

The four panels of Figure 7 present predicted RFS proportions at 5 years; each panel has identical solid lines, but in each a different nodal status group is highlighted. The two solid vertical bars in each panel compare a 7 log-kill to a 9 log-kill range (right bar), and a 7 log-kill to the 2-to-4-log-kill range (left bar). The heights of these bars are given in Table 2. Among patients with 10 or more positive axillary nodes (panel D), the increase from standard therapy to intermediate therapy (left-hand vertical bar) is substantial, and the increase from ID to high-dose therapy (right-hand vertical bar) is smaller but still of some clinical interest. In contrast, among patients with no positive axillary nodes (panel A), the smaller log kills associated with standard therapy would be sufficient to cure the smaller burdens of sensitive tumours found in most of the node-negative patients who have at least one remaining viable tumour cell postoperatively (left vertical bar in Figure 8, panel A). In particular, for patients with four to nine positive nodes (panel C), the added benefit of high-dose *vs* ID therapy (right-hand vertical bar) is minimal. Figures 6, 7 and 8 demonstrate the principle that the dependence of RFS on the log kill is steepest in the range of low log kills in patient groups with the smallest pretreatment RTBs, and steepest in the range of high log kills in patient groups with the largest pretreatment RTBs.

## DISCUSSION

The focus on RFS was driven by the goal of understanding the dynamics underlying the generation of relapses. This strategy is subject to the caution that RFS often fails to reflect patient survival. Arguments for this effect related to Gompertzian growth can be found (Dang *et al*, 2003), and early treatment-related mortality (Peters *et al*, 2000; Tallman *et al*, 2003) augments this effect. Therefore, a model that describes the occurrence of relapses accurately might well predict patient survival poorly. Nevertheless, RFS and survival are both affected by residual tumour, by regrowth, and by treatment effect, so understanding the role of these three factors better may contribute to understanding better how to extend survival.

These three factors are likely to be associated with expression of sets of genes. In explorations of gene expression as predictors of recurrence, it will be useful to keep in mind that recurrence depends on RTB, the sensitivity to the administered therapy, and the regrowth curve. Expression of specific genes may be associated with one or more of these three factors. If the factors are tightly correlated (in contradiction to our assumption), then a small set of genes may be strongly predictive, but if the independence assumption is closer to the truth, then even a gene group, which predicts one of the factors with great accuracy may still not predict recurrence well, because of variance in the other two factors. At the same time, a fourth source of individual variation, pharmacokinetics, may be just as critical. A randomised study compared marrow-supported high-dose chemotherapy with fluorouracil, epirubicin, and cyclophosphamide (FEC) ‘tailored’, that is, individually dose adjusted according to haematologic toxicity parameters. The RFS was significantly better for the FEC-tailored arm (Bergh *et al*, 2000). In contrast, a randomised study (Rodenhuis *et al*, 2003), which compared marrow-supported high-dose chemotherapy to FEC without individual tailoring, did not show significant differences in RFS at 5 years.

A potential mechanistic explanation for the results of Figures 6 and 7 can be seen in Figure 8. Panel 8A shows model-derived RFS curves for patients who either were untreated or were treated with regimens capable of 3, 7, or 9 log kills in sensitive tumours. The corresponding post-treatment residual body burden distributions are shown in panels 8B_1_–8B_4_. As an estimated ∼50% of patients have resistant tumours, successively increasing the log kill leaves a portion of the RTB distribution unchanged, even with maximal treatment (8B_4_). The notches in these distributions reflect the artificial working assumption of a sharp distinction between absolutely resistant tumours and sensitive tumours. With increasing log kill, an increasing proportion of modelled patients with sensitive tumours is rendered relapse free, as indicated by the progressive shift in the notch point in the distribution to the left. The regions in panels 8B_3_ and 8B_4_ marked by ‘x’ correspond to a small subset of patients who would experience tumour recurrences with 9 logs of tumour reduction but not with 7 logs. The time for the tumours in this subset to grow to a detectable size is substantial, affecting primarily the later portion of the RFS curve. Future, recent and forthcoming publications of randomised trials will provide the ability to check this prediction.

Our simulation studies suggest several conclusions. A substantial proportion of patients with 10+ axillary nodes, who receive adjuvant treatment with maximally tolerated systemic therapy, still develop clinical recurrences. A substantial proportion of patients may have absolutely resistant tumours. Patients with sensitive tumours and 10 or more positive nodes may benefit from treatment intensification, provided that toxicity is manageable, but much of this clinical benefit might be achieved using ID rather than HD levels of treatment intensification (Figures 6 and 7). Finally, the results suggest that patients with fewer than 10 involved axillary nodes, and therefore generally smaller RTBs, who have sensitive tumours, are more likely to be cured by standard treatment (Figures 6 and 7), and are, therefore, less likely to benefit from treatment intensification.

These predictions can be compared with the results of studies involving adjuvant breast cancer treatment intensification. Several reviews of these studies have been published (Antman, 2001; Baynes *et al*, 2001; Gianni *et al*, 2001; Rodenhuis *et al*, 2003). Early published studies of intensive adjuvant treatment regimens in postoperative breast cancer patients with 10 or more positive axillary nodes had small sample sizes or were not randomised. More recently, two large randomised multiinstitutional adjuvant treatment intensification trials have been published. For the French PEGASE 01 trial (Roche *et al*, 2003), which compares high-dose therapy with standard therapy in patients with eight or more positive axillary nodes (>150 patients per treatment arm), metastasis-free survival was significantly longer for patients who received high-dose therapy, but overall survival was not. In a Dutch study (Rodenhuis *et al*, 2003), comparing the results of intensive adjuvant therapy with those of standard therapy in high-risk patients (>440 patients in each treatment arm), only one-third of the patients had 10 or more positive axillary nodes, while two-thirds of the patients had 4–9 positive axillary nodes. This study demonstrated a statistically significant increase in RFS (but not overall survival) in patients receiving high-dose therapy, only in those with 10 or more positive axillary nodes. Thus, the RFS results of the Dutch study are consistent with our conclusions.

Tallman *et al* (2003) recently reported a study comparing standard-dose therapy with high-dose therapy in patients with 10 or more positive axillary nodes, with over 250 patients in each arm. An increase in RFS for high-dose therapy had marginal statistical significance, sensitive to inclusion of patients with minor protocol violations.

The study of Peters *et al*, which has not been published as of this writing, compares a treatment regimen of maximal intensity with an aggressive regimen of intermediate treatment intensity in patients with 10 or more positive nodes, but does not include a standard therapy arm (Peters *et al*, 1999, 2000). Each treatment arm included over 390 patients. The fractions of patients surviving relapse free after 5 years of follow-up are greater than 0.5 for both regimens, exceeding the results of any large published studies involving pure subsets of patients with 10 or more positive axillary nodes treated with conventional adjuvant treatment regimens. However, there was substantial treatment-related early mortality in the high-dose arm.

A small published randomised MD Anderson study involving 78 patients compared high-dose with conventional dose adjuvant therapy in high-risk patients, and failed to show an advantage for intensive therapy (Hortobagyi *et al*, 2000). Most patients had 10 or more positive nodes at initiation of adjuvant therapy. The study was designed to detect a true difference of 30% between the treatments in a 3-year relapse-free interval. From Figures 7 and 8, such a large difference would not be expected.

A study involving a 50% escalation of doxorubicin dosage in node-positive patients offered no therapeutic advantage (Budman *et al*, 1998). However, a doubling of anthracycline dose has been reported to produce a statistically significant improvement in RFS at 5 years in patients with 4 or more positive axillary nodes, but not in patients with 1–3 positive axillary nodes (The French Adjuvant Study Group, 2001). This is consistent with the principle emerging from our studies, which states that benefits of increasing dose should be greater for patients with greater RTB.

The National Surgical Adjuvant Breast and Bowel Project (NSABP) examined the role of three levels of intensification of cyclophosphamide alone in the AC regimen (doxorubicin plus cyclophosphamide) in operable breast cancer patients with positive axillary nodes (Fisher *et al*, 1999). There was no overall RFS benefit to treatment intensification for patients with 1–3 positive axillary nodes in that study. However, the RFS of patients with 4–9 positive nodes, who received the most intensive cyclophosphamide regimen, was significantly better than that of patients with the lowest cyclophosphamide dose administered (*P*=0.05). In this study, there were over 250 patients with 4–9 positive axillary nodes in each of the treatment arms. In contrast, there were fewer than 100 patients with 10 or more positive axillary nodes in each treatment arm. While the RFS curves of patients with 10 or more positive axillary nodes, who received the more intensive regimens, were higher than for the group that received the lowest cyclophosphamide dose, the differences did not achieve statistical significance. This study was not designed to be powered for subset analysis or interactions with nodal status.

Zander *et al* (2004) have recently published the initial results of a German trial in which standard-dose therapy with EC (epirubicin and cyclophosphamide) and CMF was compared with EC followed by high-dose therapy with CTM (cyclophosphamide, thiotepa, and mitoxantrone) in breast cancer patients with 10+ axillary nodes. The median follow-up time was 3.8 years. The event-free survival curves exhibited no discernable difference in the first 2 years, but showed increasing splay at later times. The event-free survival curve for the high-dose regimen appeared to plateau at 50%, and the event-free survival curve for the standard-dose regimen continued to decrease with longer follow-up. However, the difference was not statistically significant. As noted by the authors, the power of the study was low due to low patient accrual, and follow-up duration was too short to draw definitive conclusions.

In relation to the published randomised trials, the models presented here are broadly consistent, explanatory of some results, and point towards strategies for investigating dose issues further. Based on our findings, we would make the following recommendations for future clinical studies addressing adjuvant dose intensification in patients with operable breast cancer:

1. HD protocols should focus on patients with at least 10 positive nodes. Such patients are not only at the highest risk for recurrence but also most likely to benefit from the treatment intensification. Such studies should be powered adequately to detect differences in RFS, which are in the range of 10% or less after 5 years of follow-up. Follow-up should be extended well beyond 5 years, when the clinical outcomes of patients presumed to have sensitive tumours with small and moderate RTB's and/or substantial but noncurative log kills are likely to have their greatest effects on the RFS curve.
2. For patients with 4–9 positive axillary nodes, the predicted magnitude of benefit from ID intensification may be large enough to warrant study, while the incremental benefit from high-dose intensification may be too small to justify the excess risk of toxicity.
3. Overviews of published randomised studies of dose intensification should take into account nodal status and/or other putative correlates of RTB, for example, through subsetting patients or modelling interaction terms between treatment and the tumour burden proxy. The time is ripe for such overviews.

All models use assumptions. Most modelling of clinical data uses empirical models, with convenience assumptions such as proportional hazards. The models used here rely on assumptions with some biological content. Models with biological content can lead further, but at a risk, because the assumptions will inevitably be wrong to some degree. A famous aphorism (Box and Draper, 1987) is frequently paraphrased as ‘All models are wrong … some are useful’. This insight has a new partner (Sterman, 2002): ‘All decisions are based on models … and all models are wrong.’ Conversely, although knowledge is always incomplete, nonetheless decisions must be made. Modelling, whether formal and mathematical or informal and purely mental, takes place in the effort to plan clinical trials or understand their results. Formal modelling should improve that effort, but cautious consideration of the assumptions is demanded. It is in this spirit that our modelling investigations have been conducted.

## Figures and Tables

**Table 1:** RFS or event-free survival (^*^) in patients with 10 or more positive nodes treated with standard-dose treatment regimens

**Regimen**	**RFS at 5 years**	**No. of patients**	**Reference**
CMFVP	0.27	56	Jones *et al* (1987)
CMF	0.23	71	Jones *et al* (1987)
AC	0.30	34	Jones *et al* (1987)
FAC	0.52	66	Jones *et al* (1987)
Adriamycin-containing standard-dose regimens	0.41	283	Buzdar *et al* (1992)
CMFVP^*^	0.30	NA	Peters *et al* (1993)
CMFVP/VATH^*^	0.32	NA	Peters *et al* (1993)
AC	0.36	106	Fisher *et al* (1997)
AC, cyclophosphamide 1200 mg m^−2^	0.34	99	Fisher *et al* (1999)
FAC	0.52	42	Hortobagyi *et al* (2000)
CMF or CMF plus tamoxifen^*^	0.22	141	Schmoor *et al* (2001)
FEC	0.51	159	Rodenhuis *et al* (2003)
Tailored FEC	0.58	251	Bergh *et al* (2000)
CAF	0.52	257	Tallman *et al* (2003)
FEC	0.40	155	Roche *et al* (2003)
EC-CMF	∼0.37	129	Zander *et al* (2004)

NA=not applicable.

**Table 2:** Incremental increase in RFS at 5 years, in relation to dose intensity by axillary nodal status

	**Node negative**	**1–3 nodes**	**4–9 nodes**	**10+ nodes**
Intermediate-dose intensity (7 log kill) *vs* standard therapy (2–4 logs)	2–5%	5–10%	8–14%	10–16%
High-dose intensity (9 log kill) *vs* intermediate-dose therapy (7 log kill)	<1%	1.5%	3.5%	8%

**Table 3:** Modelling assumptions

1. Growth model	a. The growth curve model is Gompertzian.
	b. The Gompertz plateau is treated as known.
	c. The growth parameter *t*_1_ has a minimum value (*T*_g_), treated as known.
	d. *t*_1_– *T*_g_ has a lognormal distribution.
	
2. RTB model	a. RTB has a rescaled log *β* distribution from 10^−6^ to 10^9^ cells.
	b. RTB parameters are linear functions of node group.
	c. The cure threshold is treated as known.
	d. The recurrence detection threshold is treated as known.
	
3. Effect of chemotherapy	a. Tumours are either fully resistant or sensitive to chemotherapy.
	b. The log-kill hypothesis holds among sensitive tumours.
	c. Any changes in drug sensitivity over time, such as clonal selection or resistance induction, can be ignored.
	d. Any other effects of chemotherapy, such as changes in growth rates of surviving cells, antiangiogenic effects, or suppression of immune function, can be ignored.
	
4. Miscellaneous	a. The growth curve, residual body burden, and resistant/sensitive patient class are independent.
	b. Estimates of RTB and growth curve distributions obtained from an untreated sample are valid for treated patients.
	c. Estimates of the proportion of sensitive tumours obtained from the 10+ node sample are valid for other patient groups.

## Appendix A1

The assumptions are summarised in Table 3. Detailed discussion of the models, assumptions, and validations and sensitivity analyses are presented in an extensive supplement at the website www.oncotcap.pitt.edu/docs/rec urrence-overview. The computer code can be obtained by e-mail to day@upci.pitt.edu.
